# Experimental and Simulation Study of the Solvent Effects on the Intrinsic Properties of Spherical Lignin Nanoparticles

**DOI:** 10.1021/acs.jpcb.1c05319

**Published:** 2021-11-01

**Authors:** Tao Zou, Nonappa Nonappa, Mohammad Khavani, Maisa Vuorte, Paavo Penttilä, Aleksi Zitting, Juan José Valle-Delgado, Anna Maria Elert, Dorothee Silbernagl, Mikhail Balakshin, Maria Sammalkorpi, Monika Österberg

**Affiliations:** † Department of Bioproducts and Biosystems, School of Chemical Engineering, Aalto University, Vuorimiehentie 1, 02150 Espoo, Finland; ‡ Faculty of Engineering and Natural Sciences, Tampere University, Korkeakoulunkatu 6, 33720 Tampere, Finland; § Department of Chemistry and Materials Science, School of Chemical Engineering, Aalto University, Kemistintie 1, 02150 Espoo, Finland; ∥ Division 6.6, Physical and Chemical Analysis of Polymers, Bundesanstalt für Materialforschung und - prüfung (BAM), Unter den Eichen 87, D-12205 Berlin, Germany

## Abstract

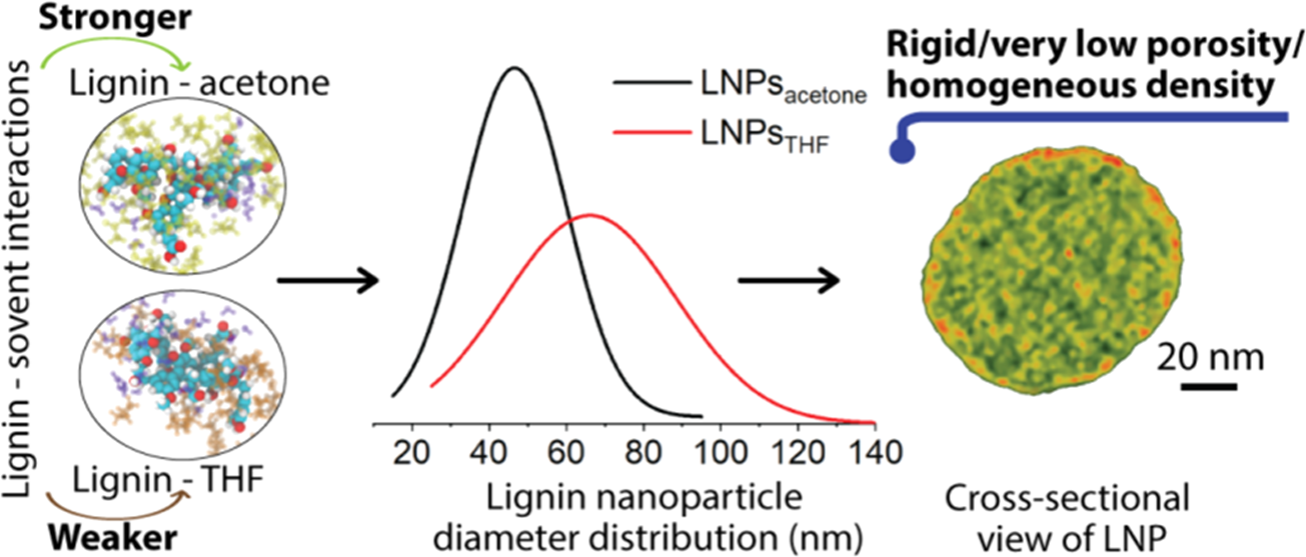

Spherical lignin nanoparticles (LNPs) fabricated *via* nanoprecipitation of dissolved lignin are among the most attractive biomass-derived nanomaterials. Despite various studies exploring the methods to improve the uniformity of LNPs or seeking more application opportunities for LNPs, little attention has been given to the fundamental aspects of the solvent effects on the intrinsic properties of LNPs. In this study, we employed a variety of experimental techniques and molecular dynamics (MD) simulations to investigate the solvent effects on the intrinsic properties of LNPs. The LNPs were prepared from softwood Kraft lignin (SKL) using the binary solvents of aqueous acetone or aqueous tetrahydrofuran (THF) *via* nanoprecipitation. The internal morphology, porosity, and mechanical properties of the LNPs were analyzed with electron tomography (ET), small-angle X-ray scattering (SAXS), atomic force microscopy (AFM), and intermodulation AFM (ImAFM). We found that aqueous acetone resulted in smaller LNPs with higher uniformity compared to aqueous THF, mainly ascribing to stronger solvent–lignin interactions as suggested by MD simulation results and confirmed with aqueous 1,4-dioxane (DXN) and aqueous dimethyl sulfoxide (DMSO). More importantly, we report that both LNPs were compact particles with relatively homogeneous density distribution and very low porosity in the internal structure. The stiffness of the particles was independent of the size, and the Young’s modulus was in the range of 0.3–4 GPa. Overall, the fundamental understandings of LNPs gained in this study are essential for the design of LNPs with optimal performance in applications.

## Introduction

Spherical lignin nanoparticles (LNPs, also called colloidal lignin particles (CLPs) or lignin nanospheres) fabricated from isolated lignins have emerged in recent years due to their advantageous features such as large surface area per unit mass, tunable surface charge, well-defined spherical shape, and colloidal stability in aqueous media in addition to the inherent properties of lignin like antioxidant and UV-screening properties.^1,2^ These features have triggered numerous studies in exploiting advanced applications of LNPs. For instance, LNPs have been demonstrated for applications in drug delivery,^3−5^ biocatalysis,^6^ antimicrobial materials,^7^ virus removing,^8^ composites,^9,10^ Pickering emulsions,^11−13^ and natural sunscreens.^14−16^

LNPs are commonly prepared by nanoprecipitation, a method that has the advantages of mild procedure, moderate equipment requirements, low energy costs, and scalability.^17−19^ In a typical nanoprecipitation process, lignin is first dissolved in an organic solvent–water mixture or water-miscible organic solvent, followed by mixing the lignin solution with a nonsolvent (usually water) that supersaturates lignin to form LNPs. In practice, mixing of lignin solution with water can either be done *via* direct dialysis using a dialysis membrane^20^ or pouring one component to the other,^12,13^ and the latter can be easier to scale up.^18^ In general, polymeric nanoparticles (NPs) prepared by nanoprecipitation are suggested to form *via* a nucleation growth process, in which the growth is dominated by random collision and aggregation of the nuclei.^21,22^ This is because nanoprecipitation results in a high number density of nuclei due to a high supersaturation level occurring over a short period (*e.g*., in seconds) that favors aggregation of nuclei over growing of the nuclei at the expense of the solute.^21^ In the case of LNPs, the formation could be more complex due to the varying molecular weight and functional groups of the raw lignin molecules.^23,24^ The current understanding is that the larger and more hydrophobic lignin molecules predominate the nucleation and aggregation, while in parallel, the smaller and more hydrophilic lignin molecules diffuse and adsorb onto the nuclei or particles driven mainly by hydrophobic and π–π interactions.^4^ The charged, hydrophilic lignin molecules at the surface play a pivotal role in providing the LNPs with electrostatic stability in the aqueous media.

LNPs prepared from nanoprecipitation usually have an average hydrodynamic diameter (*D*_h_) between dozens and hundreds of nanometers with varied polydispersity index (PDI).^2^ These parameters of LNPs strongly influence their applications or further processing, *e.g*., in drug delivery, the average size of LNPs is the key for cellular uptake,^25^ while in colloidal assembly, the polydispersity of LNPs plays a pivotal role.^26^ It has been reported that the *D*_h_ and PDI of LNPs can be tuned using different lignin sources or adjusting the preparation parameters such as the initial concentration of lignin solution, the mixing manner of lignin solution and water, and the choice of solvents. A higher-molecular-weight lignin usually results in a smaller *D*_h_,^27−30^ which could contribute to a faster nucleation rate due to a higher hydrophobicity of the larger lignin molecules. If using the same source of lignin, higher lignin solution concentration normally results in larger *D*_h_ of LNPs,^17^ probably ascribing to more formed nuclei and stronger random aggregation of the nuclei during growth. While keeping the initial lignin concentration the same, faster mixing of lignin solution and water leads to smaller *D*_h_ and PDI of LNPs,^4,18^ likely owing to a more homogeneous supersaturation and nuclei burst. In terms of solvent effects, it has been reported that the binary solvent of aqueous acetone results in smaller *D*_h_ and PDI than aqueous THF.^31^ However, the underlying reasons remain unclear. Similar phenomena have been reported earlier for NPs prepared from synthetic polymers (*e.g*., polylactides) using a pure organic solvent, that is, acetone results in smaller particles than THF.^32−34^ It has been suggested that the faster diffusion of acetone in water compared to THF promotes more uniform supersaturation leading to smaller particles.^21^ Additionally, it has been reported that the lower the solvent–nonsolvent interaction parameter and/or the higher the solvent–polymer interaction parameter, the smaller the particles prepared by nanoprecipitation.^35,36^ Acetone–water has a slightly lower interaction parameter compared to THF–water (Table 1). However, the specific interaction differences between softwood Kraft lignin (SKL)–acetone and SKL–THF remain unclear. Another important factor that affects the particle size is the level of supersaturation. It is predicted by the classical nucleation theory that a higher supersaturation level leads to a faster nucleation rate and thus a smaller particle size.^21,37^ Hence, it is important to explore the supersaturation differences of SKL in aqueous acetone and aqueous THF.

**Table 1:** Solubility Degrees of 1 wt % SKL in 75 and 21 wt % Aqueous Organic Solvents (*s*_75_ and *s*_21_) and the Corresponding Supersaturation Level (*S*), Hansen Solubility Parameter (δ), and Molar Volume (*V*) of the Organic Solvents, Solvent–SKL Interaction Parameter (χ_solvent-SKL_), Solvent–Water Interaction Parameter (χ_solvent-water_), and the Dynamic Viscosity (η) of the 21 wt % Aqueous Organic Solvent^a^

aqueous organic solvent	*s*_75_^b^	*s*_21_^b^	*S*^c^	δ of the organic solvent (MPa^0.5^)	*V* of the organic solvent (cm^3^/mol)	χ_solvent-SKL_^d^	χ_solvent-water_^e^	η of the 21 wt % aqueous organic solvent (mPa·s)
aqueous acetone	1.00 ± 0.01	0.12 ± 0.01	8.3	20.0^77^	74.1	1.6	5.6	1.3^78^
aqueous THF	1.01 ± 0.02	0.11 ± 0.01	9.2	19.4^77^	81.0	2.1	5.9	1.6^79^
aqueous DXN	1.04 ± 0.04	0.13 ± 0.01	8.0	20.5^77^	85.5	1.6	5.4	1.3^80^
aqueous DMSO	1.04 ± 0.06	0.009 ± 0.001	115.6	26.7^77^	71.0	0.01	3.2	1.4^81^

a In this context, “solvent” refers to the organic solvent and “nonsolvent” refers to water.

b Dissolved lignin concentration divided by initially added lignin concentration, average values of two replicates are shown.

c Ratio of the solubility degrees of 1 wt % SKL in 75 and 21 wt % aqueous organic solvents.

d Calculated according to ,^35^*V*_solvent_: molar volume of the solvent, Hansen solubility parameter of δ_SKL_ = 27.4 MPa^0.5^.^82^

e Calculated according to ,^36^ δ_water_ = 47.8 MPa^0.5^,^77^*T* of 298.15 K is used for all of the calculations.

In the present work, the particle sizes of LNPs resulting from aqueous acetone and aqueous THF were systematically characterized in both dry and wet states. The effect of lignin–solvent interactions on the particle size was examined using molecular dynamics (MD) simulations. The solubility and supersaturation differences of SKL in aqueous acetone and aqueous THF were compared to each other with both experimental and MD simulation results. For comparison, particle size and solvent–lignin interactions were also determined using aqueous 1,4-dioxane (DXN) and aqueous dimethyl sulfoxide (DMSO) as solvents. More importantly, the internal morphology, porosity, and mechanical properties of LNPs resulting from aqueous acetone and aqueous THF were thoroughly characterized by means of various complementary techniques. These properties have been sparsely studied previously but are relevant for fundamental understanding and various applications of LNPs.

## Experimental Section

### Materials

SKL used in this work was obtained from UPM (Finland) with the trade name BioPiva 100, which was purified from pine black liquor using the LignoBoost technology. The SKL was well characterized in previous studies.^6,38^ The sugar content of SKL was 0.05 mmol/g (determined with ^13^C NMR); the number-average molecular weight (*M*_n_) and weight-average molecular weight (*M*_w_) of SKL were 693 and 4630 g/mol, respectively (determined with gel permeation chromatography); the aliphatic, phenolic, and carboxylic hydroxyl groups of SKL were 1.89, 4.05, and 0.38 mmol/g, respectively (determined with ^31^P NMR). DMSO (ACS grade) and poly-l-lysine (PLL, 0.1 wt %, *M*_w_ = 150 000–300 000 Da) were purchased from Sigma-Aldrich. Acetone (100%), THF (99.9%), and DXN (99.9%) were purchased from VWR. All the chemicals were used as received. Deionized (DI) water was used throughout the experiments.

### Preparation of LNPs

LNPs_acetone_ and LNPs_THF_ were prepared following the same procedure described earlier.^31^ In brief, 2 g of dry SKL was dissolved in 200 g of aqueous acetone (75 wt %) or aqueous THF (75 wt %) under magnetic stirring for 3 h and the undissolved residues were removed by filtration with paper filters (Whatman, pore size 0.7 μm). Then, the solutions were poured immediately (within approximately 1 s) into vortex-stirring DI water (solution/water = 1:2.5, w/w) to form LNPs instantly. Acetone or THF was removed with dialysis in DI water using a Spectra/Por 1 tubing with an MWCO of 6–8 kDa. The final obtained concentrations of LNP_acetone_ and LNP_THF_ dispersions were 0.22 and 0.17 wt %, respectively, with a yield of above 85%.

LNPs_DXN_ and LNPs_DMSO_ were prepared following the same procedure as above. LNPs_DXN_ and LNPs_DMSO_ showed strong aggregations after dialysis; hence, the yields of these LNPs were between 70 and 80 wt % (large aggregates were removed by filtration).

### SKL Solubility Degree Measurement

The solubility degrees of SKL (1 wt %) in the 75 and 21 wt % aqueous organic solvents were measured with the mass balance method (the mass fraction of the organic solvent reduced from 75 to 21 wt % after pouring of lignin solution into water; hence, the solubility degrees of SKL in 21 wt % aqueous organic solvents were also measured). In detail, 1 wt % SKL was added into the aqueous organic solvents (75 and 21 wt %), stirred for over 3 h, centrifuged for 15 min at 13 000 rpm, and the filtered supernatants (0.2/0.45 μm syringe filters) were used for gravimetric analysis. The mean solubility degree of two replicates was used for reporting of data, which was calculated by dividing the dissolved SKL concentration over the initially added SKL concentration.

### TEM

For LNPs_acetone_ and LNPs_THF_, the samples were prepared as follows: 200-mesh Cu grids with ultrathin carbon support film (Electron Microscopy Sciences) were plasma-cleaned using a Gatan Solarus (model 950) plasma cleaner for 30 s and loaded with 10 nm fiducial gold nanoparticles as markers. Finally, 3 μL of the diluted LNP dispersions (∼0.02 wt %, diluted 10 times with DI water) were placed in the grids and the excess water was blotted using filter paper, followed by drying under ambient condition for 24 h. The images were obtained using a JEOL JEM-3200FSC field emission cryo*-*TEM operated at 300 kV in bright-field mode with an Omega-type zero-loss energy filter. The specimen temperature was maintained at −187 °C, and the images were acquired using the Gatan Digital Micrograph software.

For LNPs_DXN_ and LNPs_DMSO_, the TEM images were obtained in bright-field mode on an FEI Tecnai 12 operating at 120 kV. The samples were prepared by placing 3 μL of the dilute particle aqueous dispersion (∼0.02 wt %) on a carbon film support grid, and the excess water was removed by blotting with a filter paper, followed by ambient drying overnight.

### SerialEM and ET Reconstruction

Samples were prepared as described above for conventional TEM imaging. The collection of tilt series and processing were performed according to previously reported procedures.^39−41^ In brief, a tilt series of two-dimensional (2D) projections were acquired using the SerialEM software package.^42^ The specimen was tilted between ±69° angles using an increment step of 2–3° in low-dose mode. Prealignment, fine alignment, and cropping of the obtained tilt series were done using IMOD.^43^ To reduce noise and computation times, the images were binned two to four times. Further, reconstruction was carried out using a custom-made maximum-entropy method program^44^ on Mac cluster and a regularization parameter value of λ = 0.001. USCF Chimera was used to generate the volumetric graphics and perform image analysis.

### Cryo-TEM

For cryo*-*TEM imaging, 3 μL of the LNP dispersions (∼0.02 wt %) was applied on plasma-cleaned (30 s oxygen plasma flash) copper grids with Lacey carbon support films (300 mesh, Ted Pella, Inc.). The excess water was removed using an automatic plunge freezer (EM GP2, Leica Microsystem) with a blotting time of 3 s maintaining 90% humidity, followed by vitrification in a 1:1 liquid propane/ethane mixture. The vitrified samples were cryo-transferred to the microscope, and the cryo*-*TEM images were taken using a JEOL JEM-3200FSC field emission cryo*-*TEM (JEOL) operated at 300 kV in bright-field mode with an Omega-type zero-loss filter. The images were acquired with Gatan Digital Micrograph software, while the specimen temperature was maintained at −187 °C.

### Hydrodynamic Diameter and ζ Potential Analysis

The hydrodynamic diameter (*D*_h_) and ζ potential of the particles were analyzed with a Zetasizer Nano ZS90 instrument (Malvern Instruments Ltd., U.K.). For *D*_h_ determinization, the refractive indices (RIs) of the dispersant (water) and LNPs were set to 1.33 and 1.4, respectively, and the scattering angle was set at 90°. The mean *D*_h_ of number-based *D*_h_ distribution was used for reporting of data. For ζ potential measurement, a dip cell probe was used and the values were obtained using automatic voltage by adapting the Helmholtz–Smoluchowski equation.^45^ The samples were measured at a diluted concentration of ∼0.02 wt % (diluted 10 times with DI water), and the average values of three measurements were used for reporting of data.

### MD Simulations

For simulation purposes, three simplified lignin models (model L1, L2, and L3; see the structures in Figure 2) with seven guaiacyl units were constructed based on the recently reported knowledge of softwood (Kraft) lignin.^46−49^ The models were constructed with the principle of preserving the most important features of each lignin species type with respect to interactions with solvents. For instance, the ratios of functional groups (*e.g*., aliphatic OH, phenolic OH, carboxylic OH, and carbonyl groups, double bonds, and degree of condensation) in models L1 and L2 were represented rather accurately in match with the analytical data on SKL and milled softwood lignin (MSL), respectively,^46−49^ as this plays an important role in solvent–lignin interactions. On the other hand, *e.g*., branching is strongly underestimated in the models due to the small model molecule size (very low DP) necessitated by the modeling. Additionally, some minor moieties, *e.g*., thiol groups in SKL, were omitted or combined to a more general structure. For example, in model L1, stilbene moieties of β-5 type also represent those of β-1 type, as well as vinyl ether (double bond) and phenylcoumaran (β-5) moieties. The α-5-type structure also represents various alkyl-aromatic structures. Specifically, the molecular formulas of model L1, L2, and L3 are C_68_H_74_O_22_, C_69_H_74_O_23_, and C_68_H_78_O_23_, respectively. Model L1 contained five phenolic OH, four aliphatic OH, one carboxylic OH, and one carbonyl group; model L2 contained three phenolic OH, seven aliphatic OH, and one aldehyde group; and model L3 contained five phenolic OH, six aliphatic OH, and one carboxylic OH group.

The simulations were performed using the Gromacs v2019.5 simulations software.^50−53^ The CHARMM general force field v.4.1 was used to analogously derive parameters for the model lignins.^54−57^ Parameters for acetone, THF, DXN, and DMSO were taken from the CHARMM c36 force field,^58−62^ and water was described using the compatible TIP3P explicit water model.^63,64^ To investigate solvent–lignin interactions, initially, a single lignin molecule (model L1, L2, or L3) was centered in a simulation box of (5.316 nm)^3^ before solvation. Five different solvent systems were used for lignin–solvent interaction analysis, namely, 100% water, aqueous acetone (75 wt %), aqueous THF (75 wt %), aqueous DXN (75 wt %), and aqueous DMSO (75 wt %). The molecular numbers of the solvents for simulations were 4811 for 100% water, 954 acetone and 1031 water for aqueous acetone, 838 THF and 1132 water for aqueous THF, 753 DXN and 1227 water for aqueous dioxane, and 812 DMSO and 1164 water for aqueous DMSO. The corresponding weight percentages of the models L1, L2, and L3 in the solutions were 1.4 wt % in water, 1.7 wt % in aqueous acetone, 1.5 wt % in aqueous THF, 1.4 wt % in aqueous dioxane, and 1.5 wt % in aqueous DMSO. To access lignin–lignin interactions, two model lignin molecules were placed in the center of a cubic box with the size of (5.316 nm)^3^ at an initial separation of 10 Å (minimum atom–atom distance between the two-lignin model molecules, Figure S1). The two-lignin simulation box was then solvated using water, aqueous acetone (75 wt %), or aqueous THF (75 wt %). For both single- and two-lignin systems, the same simulation procedure described below was used.

Simulation procedure: an initial energy minimization of 50 000 steps by the steepest descent method was first performed, followed by an NVT ensemble simulation of 10 ns for initial equilibration. After this, the simulation run was continued by a 200 ns NPT ensemble MD simulation. The last 150 ns of the NPT run were analyzed for the results. The simulations employed a time step of 2 fs. The temperature was controlled by the stochastic velocity rescale thermostat with a time constant of 0.1 ps and a reference temperature of 300 K.^65^ The pressure was controlled via the Parrinello–Rahman barostat with a time constant of 2 ps and a reference pressure of 1 bar.^66^ Long-range electrostatic interactions were calculated using the PME method,^67^ while the van der Waals interactions were described using the Lennard-Jones potential and a 1.0 nm cutoff (direct cutoff, no shift). LINCS^68^ and SETTLE^69^ algorithms were used to constrain the bonds involving H atoms in the model SKL and water molecules, respectively. In determining the solvent-accessible surface area, a probe radius of 0.14 nm was used. VMD was used for the visualizations.^70^ Hydrogen bonding was assessed based on geometric criteria, where the acceptor–donor distance is less than 0.35 nm and hydrogen–donor–acceptor angle is less than 30°.

### SAXS

The particle diameter and surface areas (in dispersion state) of LNPs were measured using the Xenocs Xeuss 3.0 C device equipping with a GeniX 3D Cu microfocus source (wavelength λ = 1.542 Å) and EIGER2 R 1M hybrid pixel detector at a sample-to-detector distance of 1 m. Three samples, namely, LNP_acetone_, LNP_THF_ dispersions, and SKL solution with the concentrations of 6.7, 3.4, and 10.0 mg/mL, respectively, were prepared for analysis. The liquid samples were injected into a capillary flow cell, and the data were collected for each sample at the same spot on the glass capillary. The measured intensities were corrected for cosmic radiation, integrated azimuthally over a full circle, divided by transmitted direct beam intensity, normalized to absolute scale using a glassy carbon sample, and then background-subtracted using data measured for buffer solution corresponding to each sample (H_2_O for LNPs and 0.1 M NaOH for SKL). The corrected and background-subtracted intensities were finally scaled to units of mm^–1^ by dividing by the thickness of the capillary (1.5 mm). The magnitude of the scattering vector was defined as  with θ corresponding to half of the scattering angle.

The diameter of the LNPs was determined by fitting the SAXS intensities with an analytical model for homogeneous spheres with log-normal size distribution, as described elsewhere.^71^ For determining the specific surface area, the “invariant” *Q* was determined from the experimental SAXS intensities (*I*(*q*), in absolute units) by equation^72^
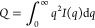
1This required extrapolating the intensity beyond its limits at high and low *q* values, which was done using the Guinier law (*I*(*q*) ∝ exp(−*R*_g_^2^*q*^2^/3)) at a low *q* and the Porod law (*I*(*q*) ∝ *q*^–4^) at a high *q* (Figure S2). The extrapolation to the low *q* is justified by previous synchrotron-SAXS data from LNPs_acetone_ prepared with the same method, which extended to smaller *q* values and showed a leveling-off behavior.^71^ The scattering length density difference between LNPs and water (Δ*ρ*) was then calculated from equation^72^
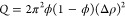
2The volume fractions ϕ and the scattering length density of the LNPs (Table S1) were calculated by assuming the scattering length density of 9.469 × 10^–6^ Å^–2^ for water. The surface-to-volume ratio *S*/*V* was determined from the SAXS intensities using equation^72^
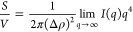
3and Δ*ρ* from eq 2. The specific surface area of LNPs in dispersion was obtained from *S*/*V* by dividing it by the volume fraction ϕ and mass density (1.4 g/cm^3^) of the particles.^73^

### AFM

A MultiMode 8 AFM equipped with a NanoScope V controller (Bruker Corporation) was used to measure the morphology and Young’s modulus of LNPs. The samples were prepared as follows: LNPs were adsorbed onto PLL-modified silicon wafer by immersing the wafer (∼1 × 1 cm^2^) in the particle dispersions (at the native concentration of ∼0.2 wt %) for 1 h, followed by rinsing with DI water and N_2_ drying. PLL-modified silicon wafer was obtained by immersing the purified wafer (15 min in a UV-ozone cleaner, Bioforce) in PLL solution (0.1 wt %) for 1 h, followed by rinsing with DI water and N_2_ drying.

The AFM images were obtained in tapping mode in ambient air using NCHV-A probes (Bruker). Nanoscope Analysis (version 1.5, Bruker) was used for image processing.

Young’s moduli of the samples were measured using Force Volume mode in ambient air. A RTESPA-525 probe (Bruker, *k*_c_: 200 N/m, *f*_0_: 525 KHz) was used for the measurements. A clean mica substrate was used to calibrate the deflection sensitivity, and a standard polystyrene substrate with a Youngs’s modulus of 2.7 GPa (Bruker) was used to calibrate the radii of the probe at different indentation depths. The tip radius was set to 50 nm, which corresponded to 2–3 nm indentation depth. The scan size was 1 × 1 μm^2^ with a resolution of 32 × 32 pixels. The indentation speed was 1 μm/s.

Nanoscope Analysis (version 1.5, Bruker) was used for data analysis. The Hertzian (Spherical) model was used for fitting the experimental data, setting the Poisson’s ratio at 0.3. A total number of 2048 indentation force curves (two images of 32 × 32 pixels) were collected for LNPs_acetone_ or LNPs_THF_. Young’s moduli were obtained by fitting the segment of the indentation force curves between 5 and 50% of the maximum applied force, which correlated well to indentation depths between 2 and 3 nm.

### ImAFM

The stiffness of LNPs was measured with an MFP-3D Asylum (Oxford Instruments Asylum Research, Inc., Santa Barbara, CA) equipped with an Intermodulation Products AB (Segersta, Sweden). The Intermodulation Products AB enables us to obtain high-resolution force spectra. The samples were prepared as described above for conventional AFM. PointProbe Plus (PPP-NCHR, Nanosnesors Neuchâtel, Switzerland) was used as the probe, which has a resonance frequency (*f*_0_) of 309.9 kHz, a spring constant (*k*_c_) of 27.18 N/m, and a quality factor (*Q*) of 487.3. The tip radius was estimated at ∼10 nm. The amplitude-dependent force spectroscopy (IM ADFS) method was adapted to obtain the force curves (1 × 1 μm^2^ with a resolution of 256 × 256 pixels) at a scan rate of 0.75 Hz in the fast scan direction.^74,75^ The indentation depth ranged from 2 to 8 nm. The 128 × 128 indentation force curves were exported for further analysis and reporting of data using SOFA.^76^

## Results and Discussion

### Solvent Effects on the Size Distribution of LNPs

LNPs were prepared from SKL using the binary solvent of aqueous acetone or aqueous THF. The mass ratio of acetone or THF to water was controlled at 3:1, because around this ratio, SKL showed the highest solubility.^17,18,23,24^ The resulting particles are termed as LNPs_acetone_ and LNPs_THF_, respectively.

**Figure 1:**
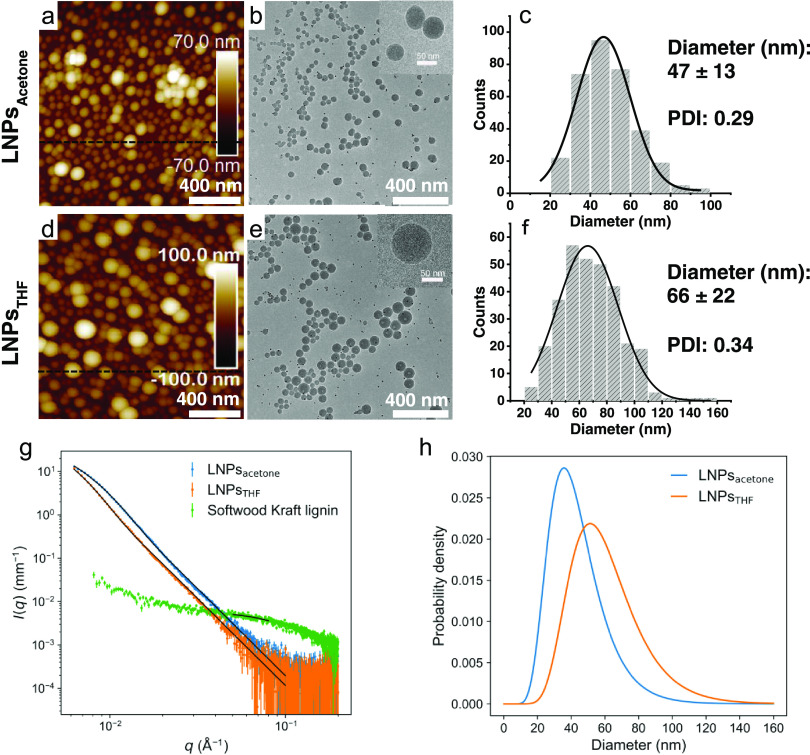
Morphologies and size distribution profiles of LNPs prepared from aqueous acetone and aqueous THF. (a–c) AFM height image, TEM image and cryo*-*TEM image (inset), and particle diameter distribution of LNPs_acetone_. (d–f) AFM height image, TEM image and cryo*-*TEM image (inset), and particle diameter distribution of LNPs_THF_. The mean diameters in (c) and (f) were calculated using Gauss Fitting Function (Origin Pro) based on over 300 particles from TEM images presented here and in Figure S3. (g) SAXS intensities of the LNP_acetone_, LNP_THF_ dispersions and SKL solution (points) and fits (solid line) of a model for solid spheres (LNPs) or Guinier law for dissolved SKL (in 0.1 M NaOH). The Guinier fit yields a radius of gyration (*R*_g_) of 1.6 nm for SKL. (h) Log-normal distributions of the particle diameters of LNPs_acetone_ and LNPs_THF_ resulting from the SAXS analysis.

In dry states, LNPs_acetone_ exhibited a relatively smaller particle size with higher uniformity in comparison to that of LNPs_THF_, as revealed by atomic force microscopic (AFM) height images and transmission electron microscopic (TEM) images (Figure 1a,b,d,e). This observation is consistent with our previous findings.^31^ We emphasize that LNPs were already present in particle form and spherical shape in the dispersion state before drying, as confirmed using cryogenic transmission electron microscopy (cryo*-*TEM) (Figure 1b,e, inset). Quantitatively, the mean diameters of LNPs_acetone_ and LNPs_THF_ were 47 ± 13 and 66 ± 22 nm, respectively, calculated based on over 300 ambient-dried particles according to TEM images (Figure 1c,f; see more TEM images in Figure S3). In addition, the polydispersity index (PDI) (standard deviation divided by mean diameter) of LNPs_acetone_ was smaller (0.29) than that for LNPs_THF_ (0.34), indicating a higher uniformity of the former. Similar mean diameters of 44 ± 16 nm for LNPs_acetone_ and 61 ± 21 nm for LNPs_THF_ were obtained by small-angle X-ray scattering (SAXS), using a model of homogeneous spheres with log-normal size distribution to analyze the data measured from LNP dispersions (Figure 1g,h). The SAXS results further support that LNPs were in particle form and spherical shape in the aqueous dispersion. The similar mean particle diameters obtained in dry (TEM) and dispersion states (SAXS) implicate that the LNPs are not sensitive to hydration/dehydration. Such “inert” nature of LNPs agrees well with our previous observation, that is, water is predominantly binding to the surface of LNPs, but not inducing swelling upon pH increase, as the hydrophilic groups are enriched on the surface of the LNPs.^31^ Dynamic light scattering (DLS) measurements also indicated a smaller particle size and higher uniformity of LNPs_acetone_ than LNPs_THF_. The number-based mean hydrodynamic diameter (*D*_h_) of LNPs_acetone_ and LNPs_THF_ were determined to be 59 ± 16 and 100 ± 30 nm, respectively (see the distribution profiles in Figure S4a). Nevertheless, the ζ potentials were similar for the two types of LNPs; the values were −34.8 mV (measured at pH 4.7) and −33.0 mV (measured at pH 4.6) for LNPs_acetone_ and LNPs_THF_, respectively (distribution profiles are reported in Figure S4b).

### Investigations on Solvent–Lignin Interactions

**Figure 2:**
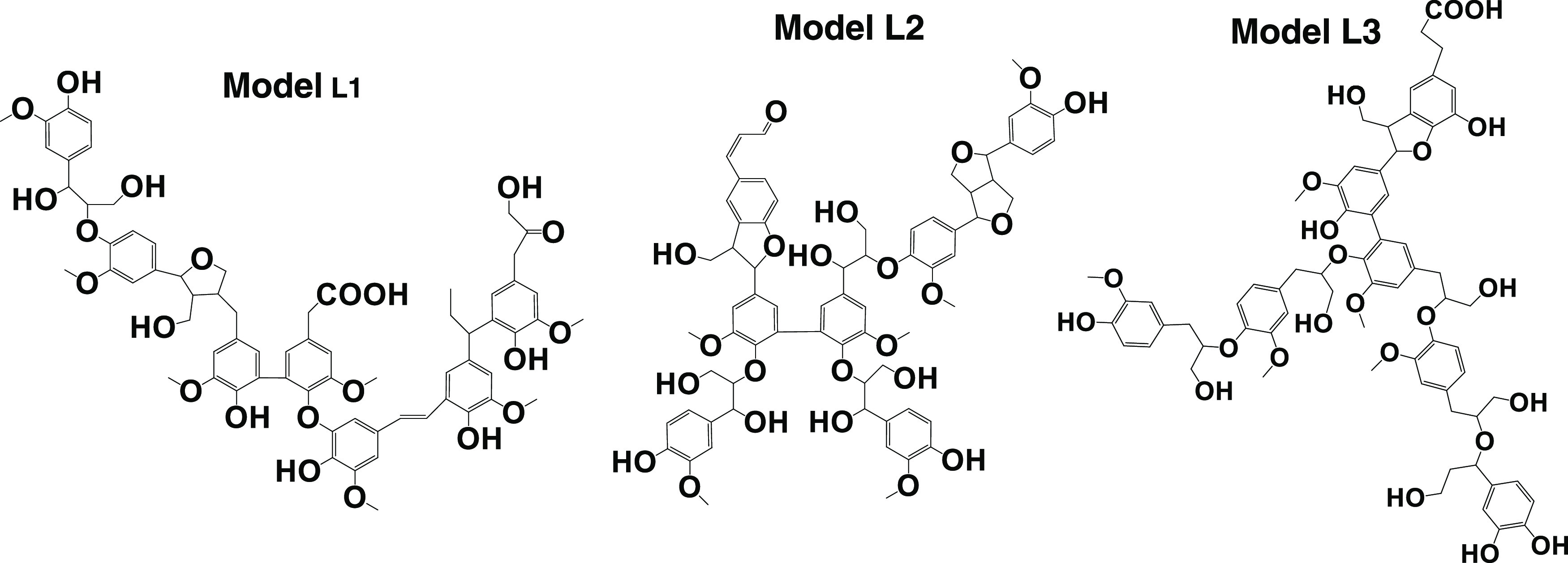
Three lignin models based on seven guaiacyl units used in the simulations as representatives of SKL structure (L1), MSL (L2), and a model with interunit linkages similar to the native softwood lignin and functional groups similar to SKL (L3).

To understand the solvent effects on the size of LNPs, MD simulations were employed to investigate the solvent–lignin interactions in aqueous acetone (75 wt %), aqueous THF (75 wt %), and pure water environments. Three lignin models, all based on seven guaiacyl units, showing varying interunit linkages and functional groups were constructed for the simulations (Figure 2). Model L1 was constructed based on the recent knowledge of the SKL structure,^46,48,49^ model L2 based on MSL,^47^ and model L3 had interunit linkages similar to the native softwood lignin and functional groups similar to SKL (see the justification of the lignin models in the Experimental Section).^46−49^ These three models were chosen to test the sensitivity of the simulation results to variations in functional groups and intermolecular linkages and to make the results more comprehensive. In all lignin model cases, the MD simulation results suggest a good solubility of the lignin model molecules in the binary solvents but a poor solubility in the pure water. This is indicated by the higher solvent-accessible surface area (SASA) of the lignin model molecule in aqueous acetone or aqueous THF compared to that in water for simulations of two-lignin molecules (Figure S5). The two-lignin model molecules aggregate in water and hence cause a reduction in SASA, whereas in aqueous THF or aqueous acetone, they are fully solvated. Experimentally, SKL (1 wt %) was almost 100% dissolved in the aqueous acetone (75 wt %) or aqueous THF (75 wt %) (Table 1) but poorly dissolved in water. The simulation results agree with the experimental observations.

**Figure 3:**
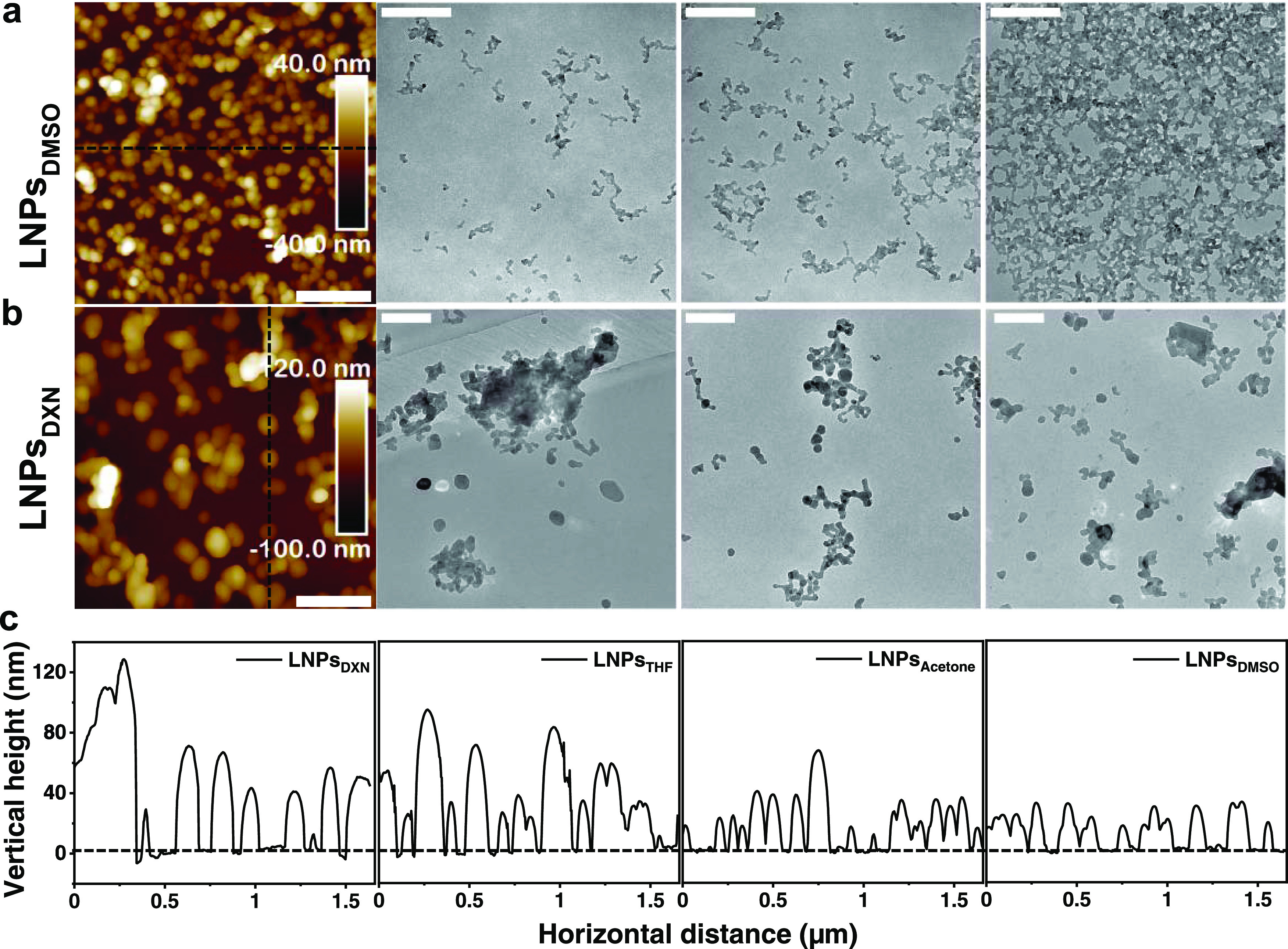
AFM and TEM images of (a) LNPs_DMSO_ and (b) LNPs_DXN_. All scale bars are 400 nm. (c) Cross-sectional height profiles of the LNPs corresponding to the black dashed lines in (a) and (b) and in Figure 1a,d. Areas with as many as possible single particles were chosen for the line scan.

We further analyzed in detail the solvent–lignin interactions in the different solvent systems. Aqueous DXN and aqueous DMSO were added for confirmation. Aqueous DXN has all of the same parameters as aqueous acetone, while aqueous DMSO shows the highest supersaturation level for SKL, and DMSO has the strongest interaction with both water and SKL among all of the studied organic solvents (Table 1). Experimentally, aqueous DMSO produced the smallest LNPs among all of the binary solvents, while the LNPs obtained from aqueous DXN showed similar particle size as LNPs_THF_ as revealed by the AFM and TEM images (Figure 3). The general LNP size order follows: LNPs_DMSO_ < LNPs_acetone_ < LNPs_THF_ ∼ LNPs_DXN_. The exact particle sizes of LNPs_DXN_ and LNPs_DMSO_ were not measured/calculated, as they aggregated extensively in both wet and dry states.

**Figure 4:**
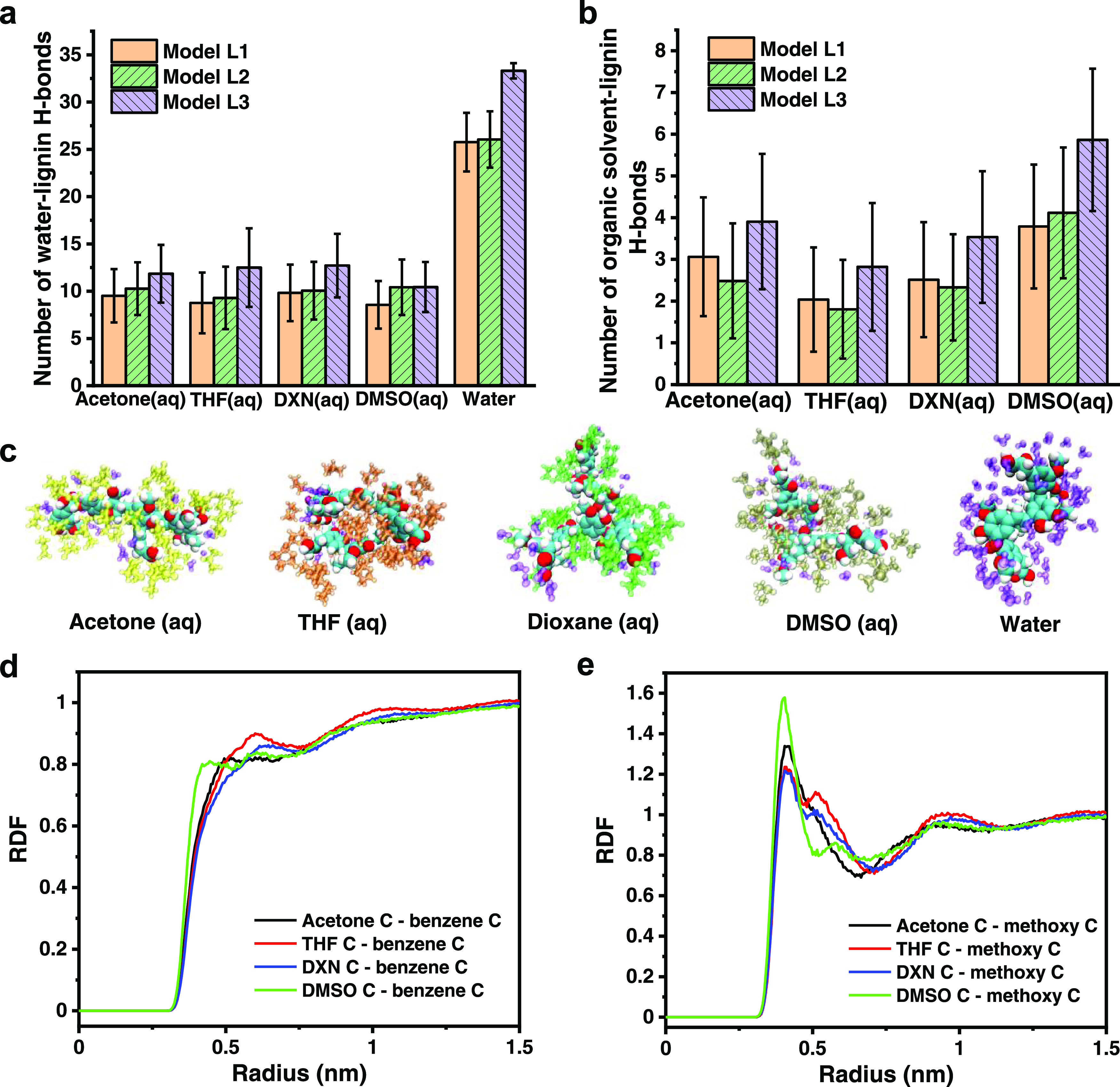
MD simulations data of the interactions between the organic solvents/water and lignin models. (a) Number of H-bonds between water and lignin models in aqueous organic solvents (75 wt %) and water. (b) Number of H-bonds between organic solvents and lignin models in aqueous organic solvents (75 wt %). (c) Visualization snapshots of the solvation of model L1 in different solvents showing displacement of water from the lignin solvation shell of 3 Å radius by organic solvents. Water is represented in the visualizations in purple, acetone in yellow, THF in brown, DXN in green, and DMSO in tan. (d, e) RDFs of the organic solvents around the hydrophobic moieties of the model L1 in the aqueous organic solvents (75 wt %). The RDFs are calculated using the C atom of the organic solvent and the C atom of (d) benzene or (e) methoxy group of the model L1. The corresponding RDFs for models L2 and L3 are reported in Figure S6.

The hydrogen bonds (H-bonds) between the solvent/water and the lignin models were first analyzed in simulation. As expected, the H-bond formation is only related to the hydrophilic groups of the lignin models. When compared, water behaves as the most efficient H-bonder as indicated by the higher number of H-bonds between water and the lignin models compared to that between the organic solvents and the lignin models in the aqueous organic solvents (Figure 4a,b). When present, the organic component replaces water in the solvation shell, as visualized by the snapshots (Figure 4c). The snapshots show that the organic solvents can also solvate the hydrophobic moieties of the lignin models, whereas water solvates only the polar groups. This explains the higher solubility of lignin in the binary solvents than in water. Detailed organic solvent–lignin hydrophobic interaction results are discussed in the next paragraph. Similar conclusions have been reported by Wang et al.,^83^ who found that water has a stronger interaction with the hydroxyl groups of a model enzymatic hydrolysis lignin (EHL) compared to acetone, based on the radial distribution function (RDF) results. In addition, when comparing the H-bonds between the organic solvents and the lignin models, the number of H-bonds had the following order: THF < DXN ∼ acetone < DMSO (Figure 4b). This order matches the solvent–lignin interaction parameter (χ_solvent-SKL_) in a reverse manner (Table 1). Previously, Choi et al.^35^ reported that the higher the solvent–polymer interaction parameter, the smaller the poly(d,l-lactide-*co*-glycolide) (PLGA) NPs prepared by nanoprecipitation. However, our results do not agree with Choi et al. in terms of experimentally observed LNP size order. We note that other parameters, including solvent–water interaction, viscosity of the binary solvent, and the supersaturation level, also contribute to the size of LNPs.^21,36,37^ However, these parameters do not fully explain the different particle sizes observed.

We hence investigated the hydrophobic interactions between the organic solvents and the benzene or methoxy group of the lignin models. The interaction distances between the C atoms of the organic solvents and the benzene or methoxy groups of the lignin models have the following order: DMSO < acetone < THF ∼ DXN (data in Figure 4d, e for model L1 and in Figure S6 for models L2 and L3). This order correlates well with the particle size of LNPs. Combining the solvent–lignin hydrophobic interaction and the solvent–lignin H-bonds results, we suggest that the stronger the solvent–lignin interactions, the smaller the LNP size. Consistent with this, Schubert et al.^22^ summarized that, for polar and aprotic solvents, the stronger affinity of the solvent toward polymer leads to smaller particle size, due to hampering of the solvent release from polymer to nonsolvent during the solvent exchange process.

Comparison of the MD simulation results on different lignin models reveals that the observed solvation trends are independent of the precise structure of the lignin model, *i.e*., the same order is followed in regard to solvent. However, the data show that the chemical structure influences the balance between hydrophilic and hydrophobic interactions: L3 formed the largest number of H-bonds, both with water and with the organic solvent species, as a result of having most free OH groups and a carboxylic acid group. For L1 and L2, H-bonding interactions were affected by both the presence of the carboxylic acid group in L1 and polarity of the OH groups.

### Internal Morphology of LNPs

**Figure 5:**
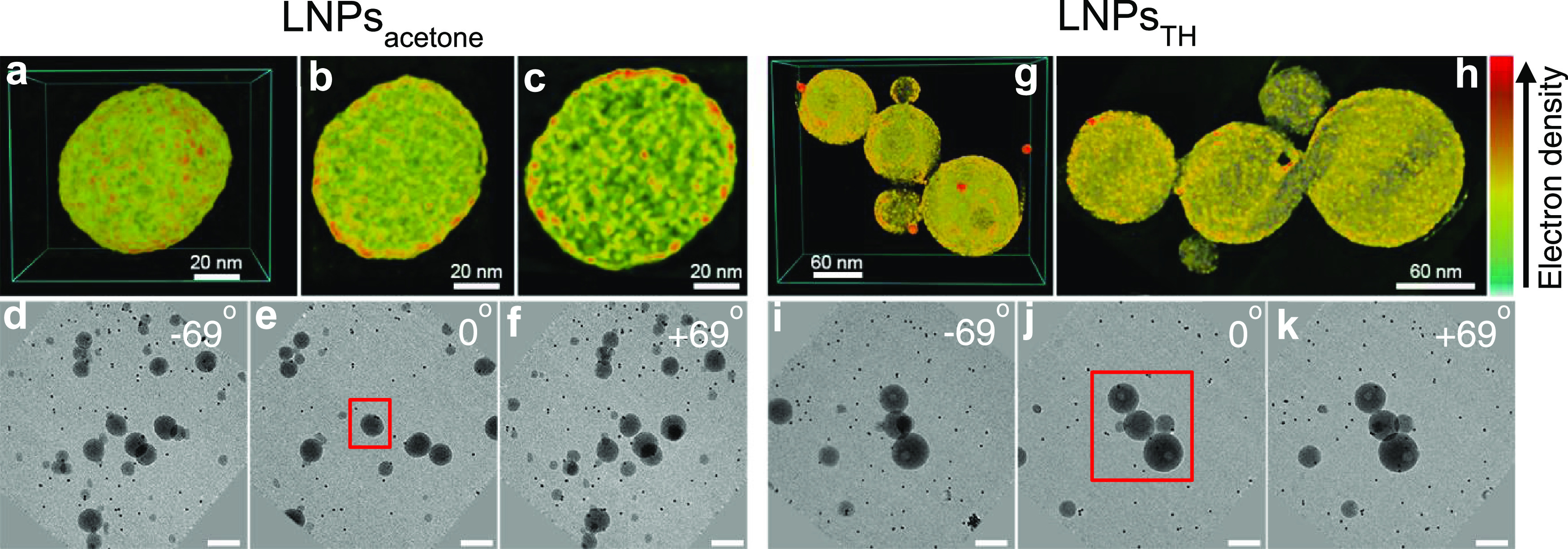
3D-reconstructed images of LNPs. (a) 3D*-*reconstructed structure, (b, c) cross-sectional views, and (d–f) the corresponding 2D TEM projections at −69, 0, and +69° extracted from the tilt series of LNPs_acetone_. (g) 3D-reconstructed structure, (h) cross-sectional view, and (i–k) the corresponding 2D TEM projections at −69, 0, and +69° extracted from the tilt series of LNPs_THF_. The scale bars in TEM projections are 100 nm. The red boxes mark the particles used for 3D reconstruction. The 10 nm fiducial gold particles were used as markers (see the red dots in (g)). Note that the electron density bar is used to indicate the density difference in one analysis, and thus the density differences of the two kinds of LNPs cannot be compared to each other.

The three-dimensional (3D) structures of LNPs and their internal morphologies were analyzed using electron tomography (ET) reconstruction. The 3D-reconstructed ET images show the spherical nature of both LNPs_acetone_ and LNPs_THF_ (Figure 5a,g). More importantly, cross-sectional views of the tomographs suggest that the spherical LNPs were composed of smaller building blocks and were rather homogeneous in density distributions (Figure 5b,c,h; see the videos in the SI). This is an important discovery, which verifies that LNPs are essentially compact rather than having a core–shell structure or being hollow despite the broad molecular-weight distribution of SKL (see the Experimental Section). However, we emphasize that in some cases, the impurities in the organic solvent or incomplete removal of the organic solvent could result in hollow LNPs. For instance, Xiong et al.^84^ reported that the analytical-grade THF (containing, *e.g*., toluene) led to hollow LNPs, whereas the chromatographic-grade THF resulted in compact LNPs, as the impurity of, *e.g*., toluene was not miscible with water that remained inside the LNPs. Li et al.^85^ used ethanol to prepare nonspherical LNPs and observed that the removal of ethanol with dialysis yielded more compact particles. In fact, in parallel to the removal of the organic solvent, the unprecipitated small lignin molecules would adsorb to the LNPs and fill the pores, as reported by Sipponen et al.^4^

### Porosity of LNPs

Inspired by the relatively homogeneous internal morphology of LNPs, we further investigated the surface areas and intraparticle porosity of the two types of LNPs using SAXS and nitrogen gas (N_2_) adsorption–desorption method. The SAXS analysis showed specific surface areas (total amount of interface between water and solid material) of 78 and 58 m^2^/g for LNPs_acetone_ and LNPs_THF_, respectively, as calculated based on the extrapolated intensities following Guinier law at a low *q* and Porod law at a high *q* (Figure S2).^86^ These values are of the same order as would be expected for monodisperse and homogeneous spheres with diameter and density similar to those of the LNPs. For example, spheres with a density of 1.4 g/cm^3^ and diameters of 44 nm (similar to LNPs_acetone_) and 60 nm (similar to LNPs_THF_) would have specific surface areas of 97 and 71 m^2^/g, respectively. This rough comparison indicates that the specific surface area determined by SAXS mainly originates from the outer surface of the LNPs. The validity of Porod law on a *q*-range from 0.02 to 0.1 Å^–1^ (Figure S2) additionally shows that the inner structure of the LNPs is homogeneous in the corresponding length scale (approximately 5–30 nm). Nevertheless, we notice that the scattering length densities (Table S1) of the LNPs obtained based on SAXS data were slightly lower than the value 12.6 × 10^–6^ Å^–2^, which would be expected for LNPs with a composition of 65.4 wt % C, 5.8 wt % H, 26.2 wt % O, 1.5 wt % S, and 0.1 wt % N and a density of 1.4 g/cm^3^.^86,87^ This difference might indicate the presence of some water molecules inside the particles, although the experimental values for the scattering length density are also sensitive to the deviations in the sample concentration as well as the assumed lignin composition and density. N_2_ adsorption–desorption results further support the solidity of LNPs. The total surface areas of LNPs_acetone_ and LNPs_THF_ in contact with N_2_ were 41 and 39.7 m^2^/g, respectively. These values are smaller than that measured by SAXS in dispersion state, probably caused by some aggregations of the particles due to freeze-drying and degassing. Moreover, the pore diameters of LNPs_acetone_ and LNPs_THF_ measured with N_2_ adsorption–desorption varied from a few to dozens of nanometers, and the former was in a smaller diameter range compared to the latter (Figure S7). These observed pore diameters originated from the interparticle but not intraparticle pores. As the reference sample, the surface area of dry SKL powder was measured to 5.2 m^2^/g and no porosity was detected (Figure S7). Previously, Sipponen et al.^71^ reported an intraparticle pore diameter of ∼5.5 nm for LNPs_acetone_, determined with LNP aqueous dispersion (0.2 wt %) using differential scanning calorimeter (DSC) following the thermoporometry-DSC (tp-DSC) method. Such a method calculates the porosity based on the freezing point depression of confined water in pores and assumes cylindrical pore geometry.^88,89^ However, the SAXS intensities from the current study do not show any indication of pores with a regular size in the scale of several nanometers, due to which we hypothesize that the confined water detected by tp-DSC could in fact be present in pores with a smaller diameter than the previously reported 5.5 nm. On the other hand, Zhao et al.^90^ recently reported the intrapore diameters of ∼1 nm and surface areas of 348–405 m^2^/g of the carbonized lignin supraparticles using N_2_ adsorption–desorption method. The lignin supraparticles used in that work were prepared by carbonization of the assembled LNPs_THF_ in the presence of 2.5 wt % cellulose nanofibrils. From these results, we can indirectly deduce that before carbonization, the intrapore diameter of LNPs is less than 1 nm.

### Mechanical Properties of LNPs

**Figure 6:**
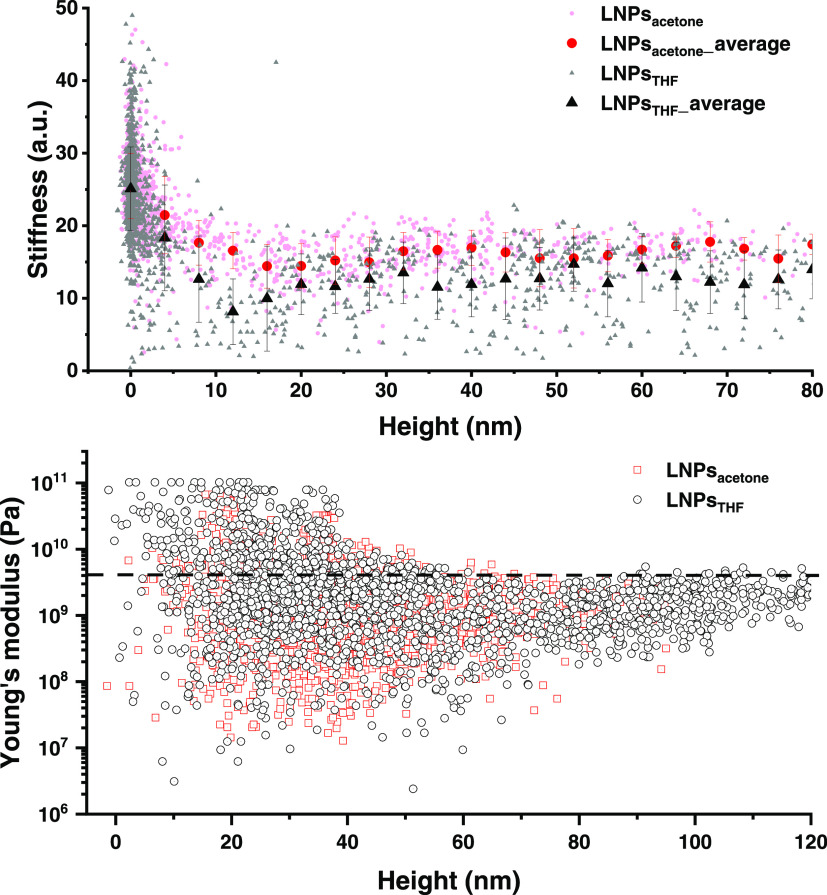
(a) Stiffness as a function of the height of LNPs_acetone_ and LNPs_THF_, measured with ImAFM. The stiffness data were taken from the very top region of the particles. (b) Young’s modulus as a function of the height of LNPs_acetone_ and LNPs_THF_ determined with AFM in Force Volume mode. The height was manually corrected to start from around 0 nm. The dashed line at 4 GPa roughly distinguishes the *E* resulting from the substrate (above 4 GPa) and the particles (below 4 GPa).

The compact structure of LNPs intrigued us to further explore the mechanical properties of LNPs, specifically their stiffness (*k*) and Young’s modulus (or elastic modulus, *E*). Our experiments by intermodulation AFM (ImAFM) revealed that the measured *k* decreased linearly from the top-middle to the top-edge of the particles (Figure S8). For a reliable correlation between *k* and particle size, the *k* values selected from the very top region of the particles (within first derivative tomography of ±0.2; see Figure S9) were plotted as a function of the particle height in Figure 6a. No significant effect of the particle size on the stiffness was observed for LNPs_acetone_ and LNPs_THF_ for particle heights between 15 and 80 nm. For heights below 15 nm, the stiffer substrate (silicon wafer coated with a thin film of poly-l-lysine) contributed significantly to the measured *k*. Interestingly, LNPs_acetone_ showed slightly higher average values of *k* than LNPs_THF_, although it must be noted that the error bars are large and partly overlap. The large error bars indicate a considerable variability between the stiffness of different particles of the same size. The variability in *k* was larger for LNPs_THF_ than for LNPs_acetone_, which might be due to more structural variability of these particles. The indentation depth is a few nanometers, so these results correspond to the properties of the corona of the particles. Nevertheless, further studies are needed to confirm this speculation. The size independence of *k* was an important finding for the LNPs, which differs from other nanospheres such as silicon or polystyrene (PS) NPs reported in the literature, for which higher *E* (proportional to *k*) was measured for smaller NPs.^91,92^ The size independence of *k* supports the solidity and homogeneous nature of LNPs regardless of the particle size.

To compare and complement the ImAFM results (which provided stiffness in arbitrary units), we measured the *E* of LNPs with conventional AFM in Force Volume mode (Figure S10). We found that the *E* values of LNPs_acetone_ and LNPs_THF_ were similar and distributed mainly between 0.3 and 4 GPa at the height above 60 nm, where there was a negligible contribution from the substrate (Figures 6b and S11). Notably, the *E* values between 0.3 and 4 GPa also included the data points measured from the off-top of the particles, due to the difficulty to select the data points only from the very top region of the particles (see Figure S10). As a result, the lower *E* boundary of 0.3 GPa could be in fact shifting to a higher value. Nevertheless, the *E* values are comparable to that of PS NPs (1–3 GPa),^91^ suggesting that LNPs could potentially replace PS NPs in many applications.

## Conclusions

In this study, we investigated the solvent effects on the intrinsic properties of spherical LNPs using the binary solvents of aqueous acetone and aqueous THF. LNPs_acetone_ exhibited a smaller average particle size with higher uniformity compared to LNPs_THF_. MD simulation results suggested that acetone had stronger interactions with the lignin models compared to THF, which contributed to the smaller size and higher uniformity of LNPs_acetone_. This was further confirmed with additional experiments with the binary solvents of aqueous DXN and aqueous DMSO. The overall interactions between solvent and lignin were insensitive to the exact structure of the lignin, but differences in functional groups lead to a different balance between hydrophilic and hydrophobic interactions. Further exploration on the intrinsic properties of LNPs_acetone_ and LNPs_THF_ revealed that both LNPs were compact particles, with rather homogeneous density distributions and very low porosities of the individual particles. Moreover, the stiffness of LNPs_acetone_ and LNPs_THF_ was independent of the particle size. Young’s moduli of the two kinds of LNPs were similar and in the range of 0.3–4 GPa. Overall, these findings shed light on the particle formation mechanism, which is valuable for the optimum design and to scale up the production of LNPs. Moreover, the detailed ET, SAXS, and ImAFM nanomechanical analysis provided valuable, novel information on the internal structure of LNPs, which is important for the application of these nanoparticles in biomedicine, adhesives, coatings, composites, and so on.

## Supporting Information Available

Initial simulation configurations of the two L3 molecules; Guinier and Porod fits for SAXS intensities measured for LNPs and table showing the corresponding scattering length density and specific surface area of LNPs obtained from SAXS; TEM images of LNPs; number-based *D*_h_ and ζ potential distributions of LNPs; SASA for lignin models in water, aqueous acetone, and aqueous THF; solvent–lignin C atom RDFs for models L2 and L3; pore diameter distributions and specific surface areas of LNPs, determined with N_2_ adsorption–desorption; stiffness plotted against the first derivative of tomography of LNPs, obtained from ImAFM results; distributions of the first derivative of tomography of LNPs, obtained from ImAFM results; Young’s modulus and the corresponding height images of LNPs, measured with AFM in Force Volume mode; and Young’s modulus distributions of LNPs, measured with AFM in Force Volume mode (PDF)
Lignin(acetone)_electron tomography (MOV)
Lignin(THF)_electron tomography (MOV)

## Supplementary Material

jp1c05319_si_001.pdf

jp1c05319_si_002.mov

jp1c05319_si_003.mov
