# Supplementary material for: Experimental and Simulation Study of the Solvent Effects on the Intrinsic Properties of Spherical Lignin Nanoparticles
Source: J Phys Chem B. 2021 Nov 1;125(44):12315–28. doi: 10.1021/acs.jpcb.1c05319 (PMC8591612; doi:10.1021/acs.jpcb.1c05319)
Supplement: Supplementary file 1 — jp1c05319_si_001.pdf [file jp1c05319_si_001.pdf]

Supporting Information for

Experimental and Simulations Study of the Solvent Effects on the Intrinsic Properties of Spherical Lignin Nanoparticles

*Tao Zou, [1] Nonappa Nonappa, [2] Mohammad Khavani, [3] Maisa Vuorte, [3] Paavo Penttilä, [1] Aleksi Zitting, [1] Juan José Valle-Delgado, [1] Anna Maria Elert, [4] Dorothee Silbernagl, [4] Mikhail Balakshin, [1] Maria Sammalkorpi [1, 3] and Monika Österberg [1,] **

[1] Department of Bioproducts and Biosystems, School of Chemical Engineering, Aalto University, Vuorimiehentie 1, 02150 Espoo, Finland

[2] Faculty of Engineering and Natural Sciences, Tampere University, Korkeakoulunkatu 6, 33720 Tampere, Finland

[3] Department of Chemistry and Materials Science, School of Chemical Engineering, Aalto University, Kemistintie 1, 02150 Espoo, Finland

[4] Division 6.6, Physical and Chemical Analysis of Polymers, Bundesanstalt für Materialforschung und –prüfung (BAM), Unter den Eichen 87, D-12205 Berlin, Germany

*Corresponding author: monika.osterberg@aalto.fi

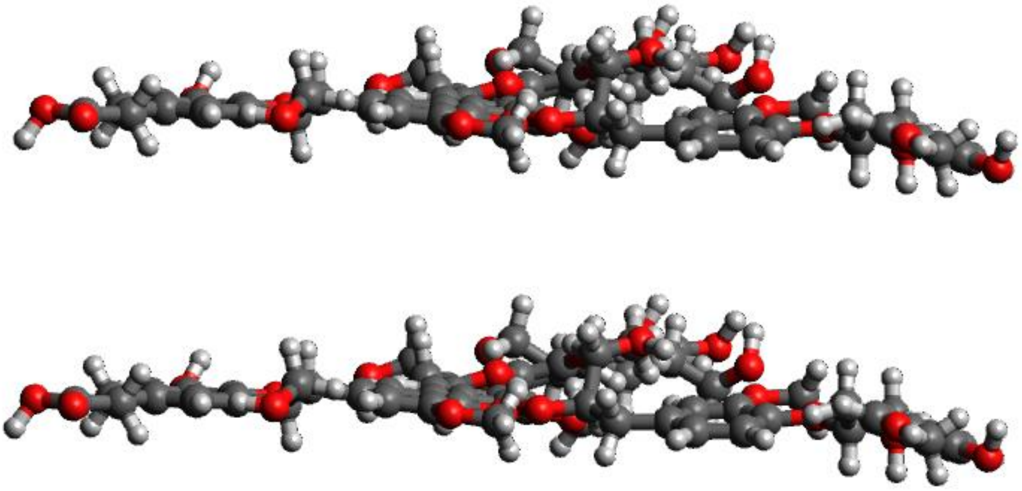

Figure S1. The initial simulations configuration of the two model L3 molecules. The shortest distance between any pair of atoms in the two molecules is 10 Å. The periodic simulations box is cubic and has a volume of $(5.316\ \text{nm})^3$.

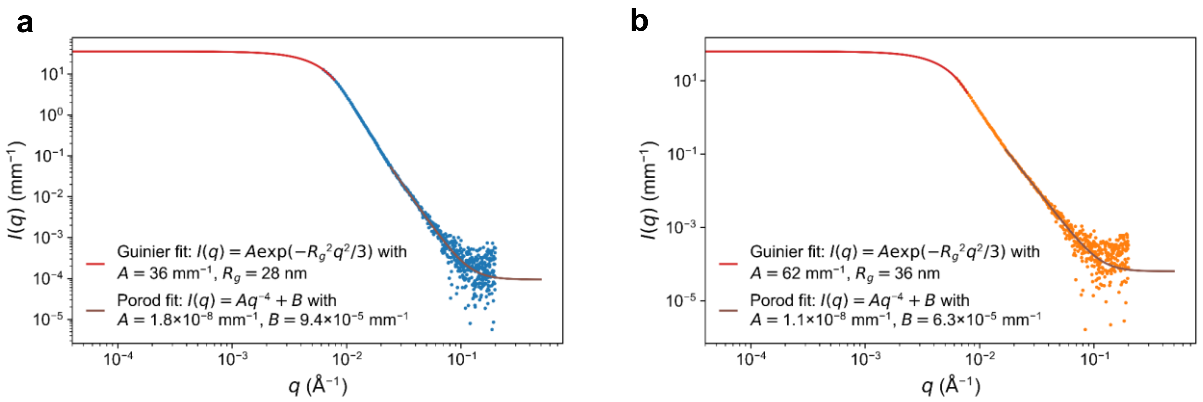

Figure S2. Guinier and Porod fits used to extrapolate the SAXS intensity beyond the measured *q*-range for (a) LNPs$_{acetone}$ and (b) LNPs$_{THF}$.

Table S1. Scattering length density and specific surface area of LNPs$_{acetone}$ and LNPs$_{THF}$ as determined by SAXS (see the fits above).

	Volume Fraction (%)[a]	Scattering Length Density (10^{-6} Å^{-2})	Specific Surface Area (m^2/g)
LNPs$_{acetone}$	0.479	11.8	78
LNPs$_{THF}$	0.243	12.4	58

[a] Calculated based on experimental concentration of the particle dispersion and a reported density of SKL (1.4 g/cm^3).[1]

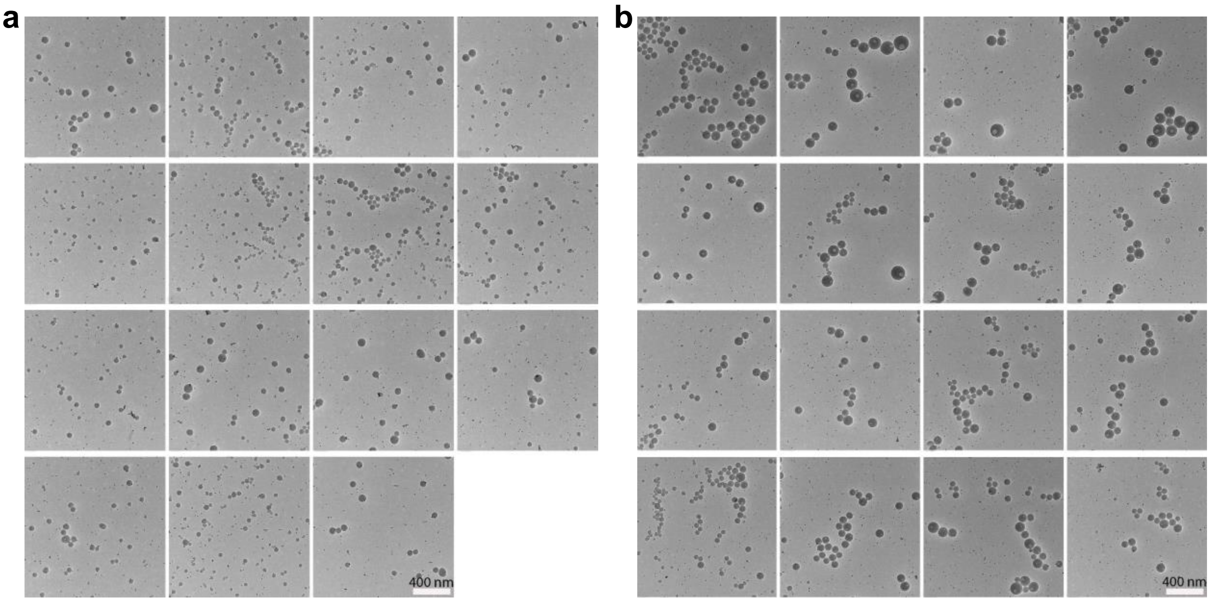

Figure S3. TEM images of (a) LNPs$_{acetone}$ and (b) LNPs$_{THF}$ that were used for determining the mean particle diameters. ImageJ Fiji was adapted for calculating the particle diameters.

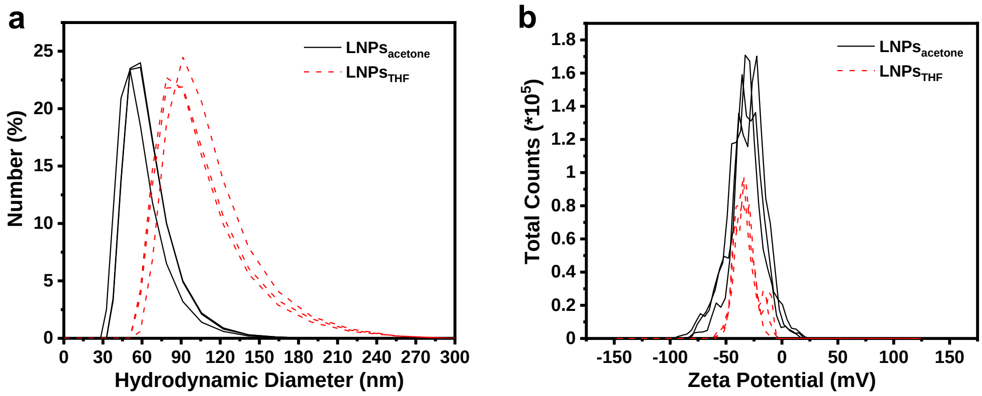

Figure S4. (a) Hydrodynamic diameter (number-based) and (b) ζ potential distributions of LNPs$_{acetone}$ and LNPs$_{THF}$.

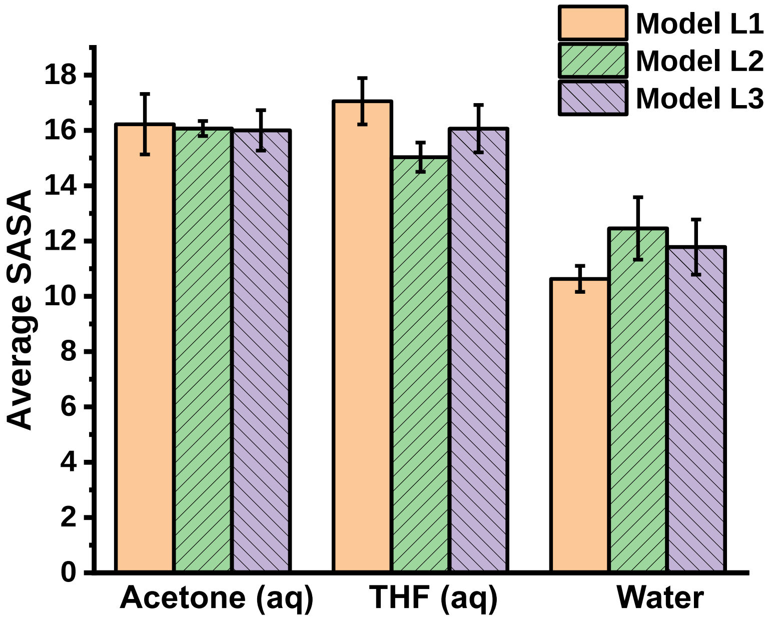

Figure S5. The solvent accessible surface area (SASA) for one lignin model in aqueous acetone (75 wt%), aqueous THF (75 wt%) and water. Note that for SASA data, simulations of two lignin molecules were used and the average values of the last 150 ns were used for reporting of data.

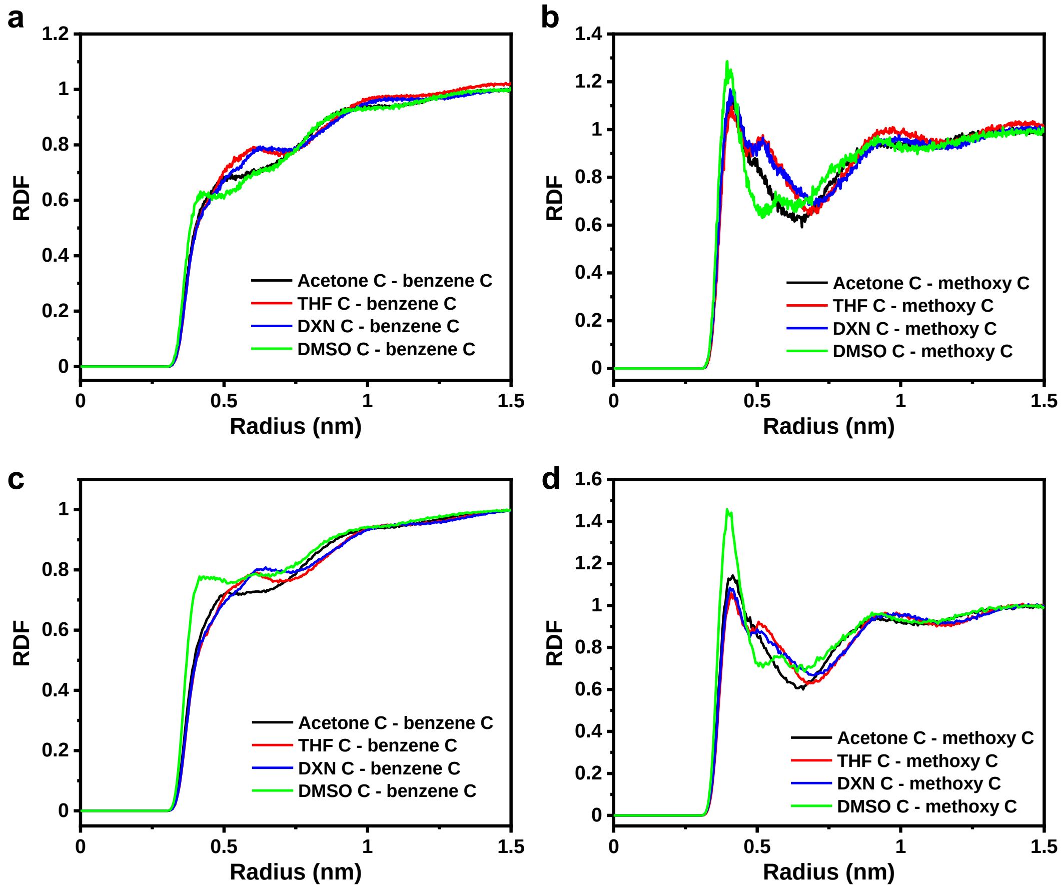

Figure S6. RDFs of the organic solvents around the hydrophobic moieties of the lignin models L2 and L3 in the aqueous organic solvents (75 wt%). Panels (a) and (b) correspond to model L2 and panels (c) and (d) to model L3. The RDFs are calculated using the C atom of the organic solvent and the C atom of benzene or methoxy group of the lignin models.

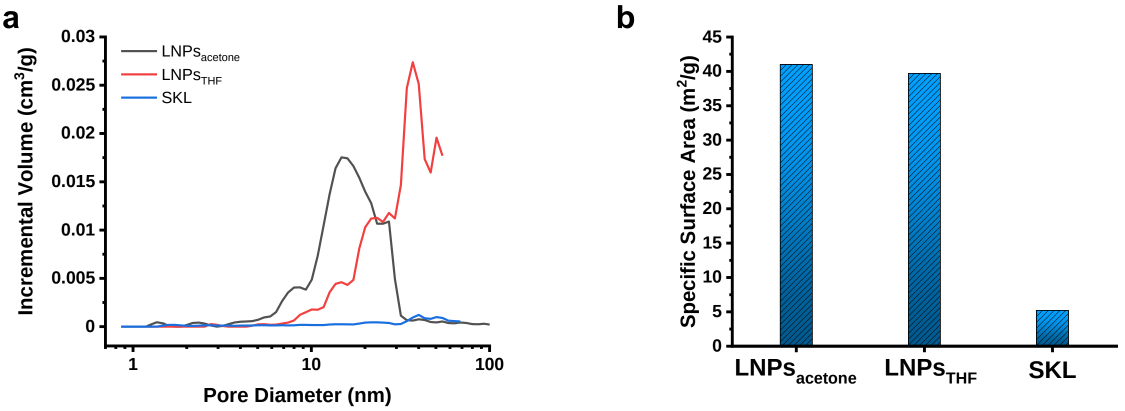

Figure S7. (a) Pore diameter distribution and (b) specific surface area of $LNPs_{acetone}$, $LNPs_{THF}$ and SKL, determined with N_2 adsorption-desorption method at 77 K (Micromeritics Tristar II equipping with an automated surface area and pore size analyzer). Prior to the N_2 adsorption-desorption measurements, the samples were freeze-dried, degassed at 105 ˚C for 12 h in the atmosphere of N_2. The Brunauer-Emmett-Teller (BET) model was adapted to determine the specific surface area (SSA), and the pore diameter distribution was obtained using density functional theory (DFT).

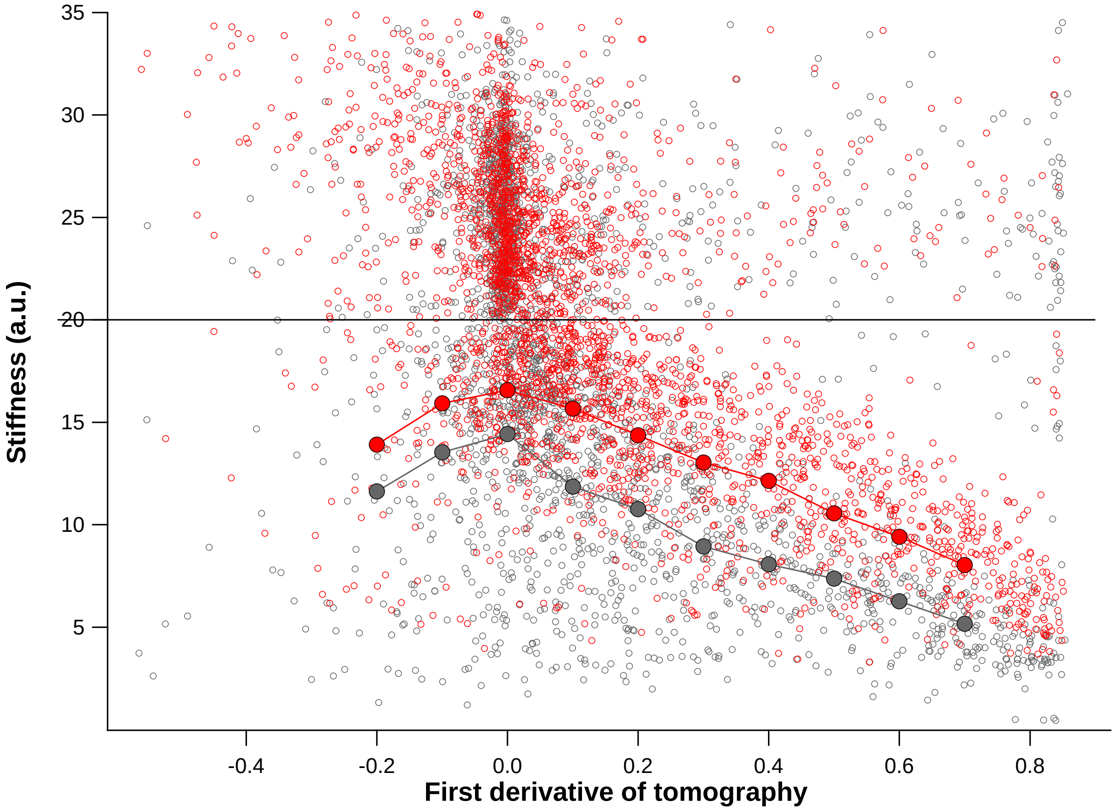

Figure S8. Stiffness plotted against the first derivative of tomography of $LNPs_{acetone}$ (red) and $LNPs_{THF}$ (black), obtained from ImAFM results.

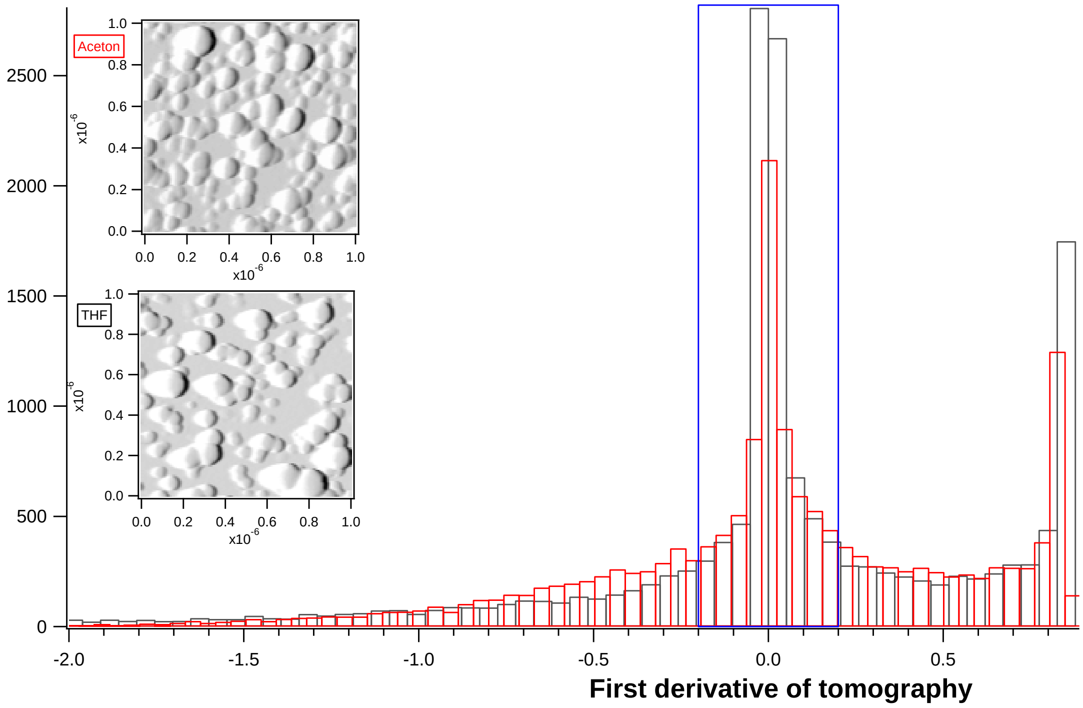

Figure S9. Distribution of the first derivative of tomography of $LNPs_{acetone}$ (red) and $LNPs_{THF}$ (black), obtained from ImAFM results. The marked area (between ± 0.2) corresponds to the very top region of the particles.

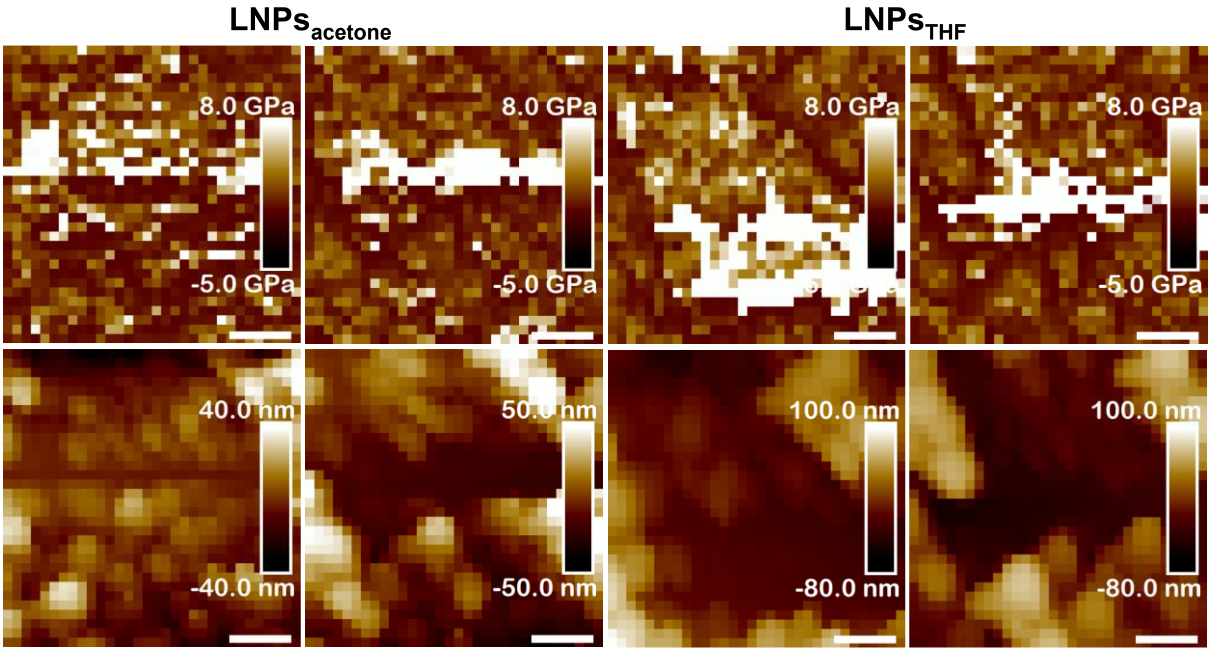

Figure S10. Young's modulus (first row) and the corresponding height profiles (second row) of $LNPs_{acetone}$ and $LNPs_{THF}$, measured with AFM in Force Volume mode. Image resolution: 32 x 32 pixels. Scale bar: 200 nm.

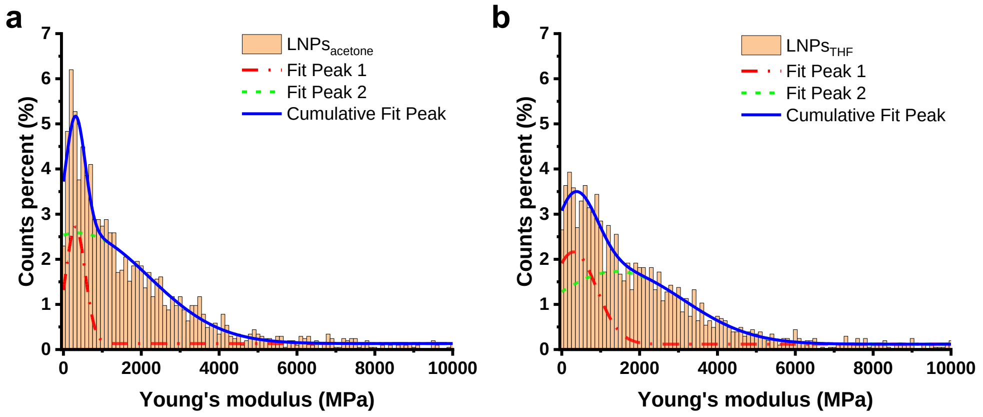

Figure S11. Young's modulus distributions of (a) LNPs$_{acetone}$ and (b) LNPs$_{THF}$ measured with conventional AFM, two peak-Gauss function was used for fitting (data above 10 GPa were excluded due to strong contribution from the substrate). The fitting resulted in the peak centers of 310 and 365 MPa for LNPs$_{acetone}$, and 302 and 1400 MPa for LNPs$_{THF}$.

Reference

(1) Vainio, U.; Maximova, N.; Hortling, B.; Laine, J.; Stenius, P.; Simola, L. K.; Gravitis, J.; Serimaa, R. Morphology of Dry Lignins and Size and Shape of Dissolved Kraft Lignin Particles by X-Ray Scattering. *Langmuir* **2004**, *20* (22), 9736–9744. https://doi.org/10.1021/la048407v.
